# The Galloyl Catechins Contributing to Main Antioxidant Capacity of Tea Made from *Camellia sinensis* in China

**DOI:** 10.1155/2014/863984

**Published:** 2014-08-28

**Authors:** Chunjian Zhao, Chunying Li, Shuaihua Liu, Lei Yang

**Affiliations:** ^1^Key Laboratory of Forest Plant Ecology, Ministry of Education, Northeast Forestry University, Harbin 150040, China; ^2^College of Resources and Environmental Sciences, China Agricultural University, Beijing 100193, China

## Abstract

Total polyphenol content, catechins content, and antioxidant capacities of green, dark, oolong, and black teas made from *Camellia sinensis* in China were evaluated. The total polyphenol content of 20 samples of tea was in the range of 7.82–32.36%. Total catechins content was in the range of 4.34–24.27%. The antioxidant capacity of tea extract was determined by the oxygen radical absorbance capacity (ORAC) test and the 1,1-diphenyl-2-picrylhydrazyl (DPPH) radical-scavenging test. Total polyphenol content, catechins content, and antioxidant capacity decreased in the following order: green > oolong > black > dark tea. A positive correlation existed between the antioxidant capacity and total polyphenol content or catechins content (*R* ^2^ = 0.67–0.87). The antioxidant capacities of five major catechins (epigallocatechin gallate (EGCG), epicatechin gallate (ECG), epicatechin, epigallocatechin, and catechin) were determined by online HPLC DPPH radical-scavenging; the antioxidant activity of tea was mainly attributed to the esterified catechins (EGCG or ECG).

## 1. Introduction

Tea consumption has a long history of more than 2000 years. Originating in China and then spreading to Japan, Europe, and other areas, tea has become one of the most popular and frequently consumed beverages in the world [1]. All kinds of tea originate from* Camellia sinensis*, which has the two subspecies var.* sinensis* (China tea) and var.* assamica* (Assam tea) [2].

In China, six types of tea made from the leaves of* Camellia sinensis* including green, black, oolong, dark, white, and yellow tea have been classified based on the degree of fermentation and the color of the tea product [1]. Green and yellow teas are nonfermented tea, and oolong and white teas are called semifermented tea, while dark and black teas are fully fermented [3, 4].

Of the six types of tea, four types (green, oolong, black, and dark teas) are widely consumed throughout the world. The manufacturing processes used to prepare the four common types of tea are as follows.

(1) Green tea: after the fresh tea is picked, it is allowed to wither for several hours. The tea leaves are then heated to remove moisture and to denature the enzymes that cause oxidation. The leaves are then kneaded and rolled while being dried. (2) Oolong tea: after the fresh leaves are picked, they are left out in the sun to wither for a few hours. Then the leaves are brought indoors and are laid out on large bamboo trays in a temperature-controlled room to oxidize. When the tea has reached the desired level of oxidation, the tea is exposed to heat to stop the oxidization. The tea is then shaped and roasted to dry. (3) Black tea: the fresh leaves are picked and allowed to wither in the sun. Once they have withered enough, the leaves are then bruised to cause oxidation. The leaves are then put into boxes to oxidize. When the leaves have turned to a dark red-copper color, they are fried and then rolled and shaped. (4) Dark tea: when the leaves are picked, they are fried, kneaded, and twisted, but instead of being dried as green tea is, the leaves are sprinkled with water and placed in huge piles under cloth to ferment before being dried. The resulting leaves have a dark black color [5].

Green tea is popular in China, Japan, India, and in Middle Eastern countries; oolong tea is mainly consumed in China, Japan, and Southeast Asia; dark tea is consumed in the southwestern regions of China; black tea is preferred in many countries throughout the world.

Recent studies have recognized that tea has health-protecting benefits that include antioxidative [6–8], antimicrobial [9, 10], antiviral [11, 12], and antitumor [13–16] activities and abilities to prevent diabetes [17], obesity [18], leukemia [19], Parkinson's disease [20], and cardiovascular disease [21].

The radical-scavenging and antioxidant properties of tea polyphenols are frequently cited as important contributors to the above-mentioned health-protection benefits [22]. Nowadays, the content of tea polyphenols is regarded as a quality indicator of tea [23]. Therefore, it is desirable to investigate the total polyphenol contents and antioxidant capacities of different teas.

**Figure 1:**
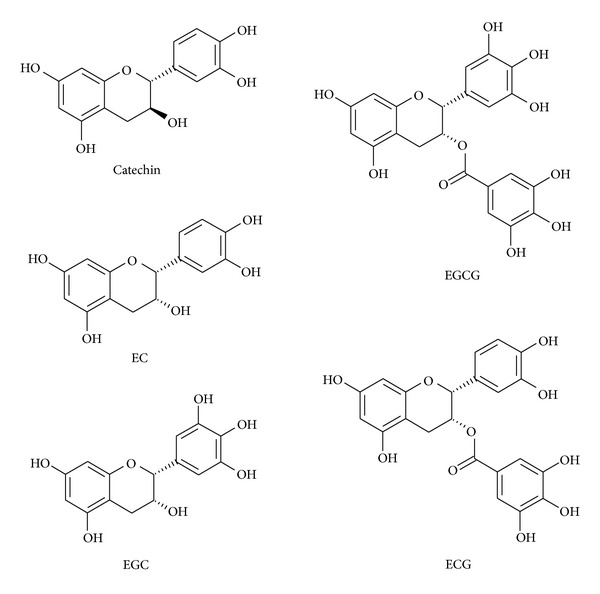
Chemical structures of major catechins present in tea.

Catechins are the most important group of active constituents in tea polyphenols. The major catechins are epigallocatechin gallate (EGCG), epicatechin gallate (ECG), epicatechin (EC), epigallocatechin (EGC), and catechin (molecular structures are shown in Figure 1) [24].

Many researchers have already begun to pay attention to the correlation between antioxidant capacity and the tea polyphenol content. Anesini et al. investigated the total polyphenol content and antioxidant capacity of commercially available tea (*Camellia sinensis*) in Argentina [25]. In their work, a high correlation was demonstrated between the two scrutinized properties. The antioxidant activity and phenolic profile in tea and herbal infusions were studied by Atoui et al. and their results suggested that black Ceylon tea and Chinese green tea infusions can be major sources of polyphenols that exhibit important antioxidant behavior [26]. Rusak et al. investigated the phenolic content and antioxidative capacity of green and white tea extracts [24]. The results showed that the antioxidative capacity of the investigated tea extracts correlated with the phenolic content. However, in the above-mentioned studies, the profiles of catechins in tea were not elucidated and it remains unclear which compounds are responsible for their antioxidant property.

In the present study, total polyphenol content and antioxidant capacity of 20 samples from four types of tea (green, dark, oolong, and black teas) prepared from* Camellia sinensis* in China were determined and the correlation between the antioxidant capacity and total polyphenol content or catechins was investigated. Furthermore, the content of individual catechins (EGCG, EGC, ECG, EC, and catechin) and their contribution to the antioxidant capacity of tea were elucidated.

## 2. Materials and Methods

### 2.1. Tea Samples

A total of 20 samples from four types of tea (green, dark, oolong, and black teas) were collected from Dafa tea market in Harbin, China. They included 5 green teas, 5 oolong teas, 5 black teas, and 5 dark teas. Their identities are listed in Table 1.

### 2.2. Chemicals

For the determination of the total phenolic content (TPC), Folin-Ciocalteu phenol reagents (Sigma-Aldrich, USA), gallic acid (GA, 97.5% purity; Sigma-Aldrich, USA), and anhydrous sodium carbonate (99% purity, Beijing Chemical Reagents, China) were used. For the determination of the antioxidant activity, 1,1-diphenyl-2-picrylhydrazyl (DPPH; Sigma-Aldrich, USA) was used. The oxygen radical absorbance capacity (ORAC) reagents of 2,2′-azobis(2-methylpropionamidine) dihydrochloride (AAPH), 6-hydroxy-2,5,7,8-tetramethylchromane-2-carboxylic acid (trolox), and fluorescein sodium were obtained from Sigma-Aldrich (USA). For the determination of the total catechins content, (−)-epigallocatechin gallate (EGCG, ≥97.0%), (−)-epicatechin gallate (ECG, ≥98%), (−)-epigallocatechin (EGC, ≥95%), (−)-epicatechin (EC, ≥98%), and (+)-catechin (≥99%) standards were obtained from Sigma-Aldrich (USA). Caffeine (≥98%) was obtained from the National Institutes for Food and Drug Control (Beijing, China). Acetonitrile, acetic acid, and EDTA were of HPLC grade (Sigma-Aldrich, USA). Methanol was of analytical grade (Beijing Chemical Reagents, China). Deionized water was prepared using a Milli Q-Plus system (Millipore, Germany).

### 2.3. Preparation of Tea Extract

The preparation of the tea extract was performed according to the method published by the International Organization for Standardization (ISO) [23]. Each tea sample was ground to a fine powder and 1.0 g of the powder was extracted with 25 mL of methanol/water (7 : 3, v/v) in a water bath at 70°C for 10 min. The extraction was repeated twice and the extracts were combined. After filtration through a filter paper and a 0.45 *μ*m membrane filter (Millipore), the extract volume was adjusted to 100 mL.

### 2.4. Determination of Total Polyphenols by Folin-Ciocalteu Method

The total polyphenol content in 20 samples from four types of tea (green, dark, oolong, and black) was determined according to the Folin-Ciocalteu method as described by ISO 14502-1 [23] with some minor modifications. One milliliter of the tea extract was diluted with water to 100 mL. The diluted extract solution (1.0 mL) was then mixed with 5.0 mL of 50% Folin-Ciocalteu reagent. After 5 min, 4.0 mL of 7.5% Na_2_CO_3_ solution was added and mixed. Then the mixture was incubated at room temperature for 60 min. The absorbance of the solution was measured at 765 nm with a Shimadzu UV-2550 UV-Vis spectrophotometer against a reagent blank. The determination was three times. The total polyphenol content was expressed as gallic acid equivalents (GAE, units: g/100 g sample).

### 2.5. Determination of Catechins by HPLC

The content of catechins in 20 samples from four types of tea (green, dark, oolong, and black teas) was determined according to the ISO 14502-2 method [27]. Quantification of the major catechins (EC, EGC, ECG, EGCG, and catechin) was performed using an HPLC system (Jasco, Japan) equipped with a 1580 pump and a 975 UV-Vis detector. Chromatographic separation was achieved with a HiQ Sil C_18_V reversed phase column (250 mm × 4.6 mm i.d.) packed with 5 *μ*m particles and the column temperature was maintained at 35°C. Mobile phase A was a mixture of 9% acetonitrile and 2% acetic acid with 20 *μ*g/mL EDTA. Mobile phase B was a mixture of 80% acetonitrile and 2% acetic acid with 20 *μ*g/mL EDTA. Baseline separation of EC, EGC, ECG, EGCG, catechin, gallic acid, and caffeine was achieved with a binary gradient elution program as follows: 100% mobile phase A for 10 min, then over 15 min a linear gradient to 68% mobile phase A, 32% mobile phase B and hold at this composition for 10 min. The detection wavelength was 278 nm and the flow rate was 0.8 mL/min. One milliliter of tea extract was diluted with water to 100 mL, the diluted extract was filtered through a 0.45 *μ*m membrane filter (Millipore), and the filtrate was injected into the HPLC apparatus.

Standard solutions of the major catechins (EC, EGC, ECG, EGCG, and catechin), gallic acid, and caffeine were prepared by accurately weighing standard powders and dissolving them in 10% acetonitrile with 500 *μ*g/mL of EDTA. The stock solutions were stored at 4°C. All standard solutions were filtered through a 0.45 *μ*m membrane filter (Millipore) and the filtrate was injected. The total catechins content was expressed as a percentage by mass of the sample on a dry matter basis and was obtained by summation of the EGC, catechin, EC, EGCG, and ECG content.

### 2.6. Oxygen Radical Absorbance Capacity (ORAC) Assay

The oxygen radical absorbance capacity (ORAC) method is used to measure the antioxidant capacity of biological samples in vitro. The ORAC assay was first developed by Cao et al. in 1993 [28]. This approach has been recognized and is now frequently used in analysis of polyphenol antioxidant capacity. 2,2′-Azobis(2-amidinopropane) dihydrochloride (AAPH) is thought to produce the peroxyl radical upon heating, which damages the fluorescent molecule and results in a loss of fluorescence [29, 30]. The fluorescence decay is monitored as fluorescein in the solution decomposes, a process that should be slower in the presence of antioxidants. Decay curves are recorded and the area between the two decay curves (with or without antioxidant) is calculated. Calculating the area under the fluorescence decay curve (AUC) of trolox, which is used as a standard antioxidant, a standard curve is prepared that can be used to evaluate the radical-scavenging activity of other antioxidants in terms of trolox equivalents (TE).

The ORAC assay was performed using a fluorescence spectrophotometer (F-7000, Hitachi, Japan), following a previously described procedure [31] with minor modification. Samples were diluted with sodium phosphate buffer (75 mM, pH 7.4). Sodium fluorescein solution (600 *μ*L, 40 nM) was added to 100 *μ*L of phosphate buffer (blank), to 100 *μ*L aliquots of trolox solution (1.25, 2.5, 5, 10, 20, 40, and 80 *μ*M), or to 100 *μ*L of diluted sample. The respective mixtures were incubated for 15 min at 37°C. Immediately before initiating the measurements, 100 *μ*L of AAPH (150 mM) was added into the test solution. With the excitation filter set to 485 nm and the emission recorded at 535 nm, readings were collected every minute for 60 min. The results were calculated based on the differences in AUC between the blank and the sample and were expressed as in units of micromoles TE per 1 g of dry tea. The AUC was calculated as follows:

(1) $$\begin{array}{c} \text{AUC}=\frac{f_{0}+2\sum_{i=1}^{59}f_{i}+f_{60}}{2f_{0}}, \end{array}$$

where *f*_0_ is the fluorescence reading at initiation of the reaction, *f*_*i*_ is the fluorescence reading at *i* minutes, and *f*_60_ is the final measurement. Net AUC was calculated as follows:

(2) $$\begin{array}{c} \text{Net}\;\;\text{AUC}={\text{AUC}}_{\text{sample}}-{\text{AUC}}_{\text{blank}}. \end{array}$$

### 2.7. DPPH Radical-Scavenging Activity

The antioxidant capacity in 20 samples from four types of tea (green, dark, oolong, and black teas) was determined by reduction of the 2,2-diphenyl-1-picryhydrazyl (DPPH) radical according to the published method [32]. A stock solution was prepared by stirring 75 mg of DPPH in 1 L of methanol overnight. A 0.75 mL aliquot of sample extract was mixed with 1.5 mL of DPPH solution. For each extract, a blank of 1.5 mL of methanol was used without DPPH reagent to correct for any sample absorbance at 521 nm. Sample and blank absorbance were recorded at 521 nm after 6 min. The percentage inhibition of DPPH was calculated according to the equation below [33]:

(3) $$\begin{array}{c} \text{Inhibition}\;\;\text{of}\;\;\text{DPPH}\;\;\left(\%\right)=\frac{\left[A_{0}-\left(A_{1}-A_{s}\right)\right]}{A_{0}}\times 100, \end{array}$$

where *A*_0_ is the absorbance of the DPPH solution, *A*_1_ is the absorbance of the tea extract in the presence of DPPH solution, and *A*_*s*_ is the absorbance of the tea extract solution without DPPH. Each sample was analyzed in triplicate.

The EC50 value was calculated by a graphical method as the concentration that caused 50% inhibition of DPPH. The anti-DPPH efficiency (AE) was also calculated as log⁡· (1/EC50) [34].

### 2.8. Online HPLC DPPH Screening Analysis of Tea Samples

**Figure 2:**
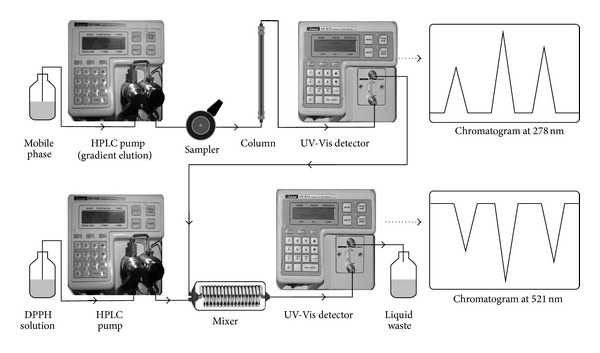
Instrumental setup for the HPLC analysis of radical-scavenging compounds using an online reaction with 1,1-diphenyl-2-picrylhydrazyl (DPPH).

The antioxidant activity of EC, EGC, ECG, EGCG, catechin, gallic acid, and caffeine was measured using the online HPLC DPPH screening method [35]. The online HPLC DPPH method was developed using a methanolic solution of DPPH stable free radical (50 mg/L). The instrumental setup is depicted in Figure 2. The flow of HPLC-separated analytes and DPPH solution was 0.8 mL/min and 0.2 mL/min, respectively. The HPLC-separated analytes reacted with the DPPH solution in post-column mode, which was photometrically detected as a negative peak at 521 nm. The length of the capillary (5.1 m × 0.5 mm i.d.) used for the postcolumn reaction was adjusted to achieve a reaction time of 1.0 min. The HPLC conditions were the same as described above.

## 3. Results and Discussion

### 3.1. Total Polyphenol and Catechins Content in Four Types of Tea

Using the Folin-Ciocalteu method, total polyphenol contents in 20 samples from four types of tea were calculated from the standard curve for gallic acid, ranging from 10 to 50 *μ*g/mL (*y* = 0.183*x* − 0.025, *R* ^2^ = 0.9987). The results are summarized in Table 2. The contents of catechins in 20 samples from four types of tea were determined by HPLC. The retention times were 7.3 min for EGC, 9.5 min for catechin, 15.3 min for EC, 16.5 min for EGCG, and 22.1 min for ECG. At the same time, the contents of gallic acid and caffeine were also determined; the retention times were 3.7 min for gallic acid and 12.4 min for caffeine. The working calibration curves were as follows: gallic acid, *y* = 208401*x* − 18547 (*R* ^2^ = 0.9996, 5–25 *μ*g/mL); EGC, *y* = 2645*x* − 976 (*R* ^2^ = 0.9989, 100–300 *μ*g/mL); catechin, *y* = 7999*x* − 1075 (*R* ^2^ = 0.9991, 50–150 *μ*g/mL); caffeine, *y* = 19823*x* − 1762 (*R* ^2^ = 0.9987, 50–150 *μ*g/mL); EC, *y* = 18574*x* − 1653 (*R* ^2^ = 0.9990, 50–150 *μ*g/mL); EGCG, *y* = 29722*x* − 2678 (*R* ^2^ = 0.9992, 100–400 *μ*g/mL); ECG, *y* = 16774*x* − 1546 (*R* ^2^ = 0.9988, 50–200 *μ*g/mL), where *y* is the peak area of the analyte and *x* is the concentration (*μ*g/mL). The results for the catechins are summarized in Table 2. It can be seen from Table 2 that the content of total polyphenols ranged from 7.82 to 32.36 g of gallic acid equivalent/100 g dry tea and the content of catechins ranged from 4.34% to 24.27% (dry tea). Of the selected 20 tea samples,* Bi luo chun* (green tea) had the highest content of polyphenols and catechins. Although there was a significant difference in the content of polyphenols and catechins in the same type of tea, as a whole, the contents of total polyphenols and catechins decreased in the following order: green tea > oolong tea > black tea > dark tea.

**Figure 3:**
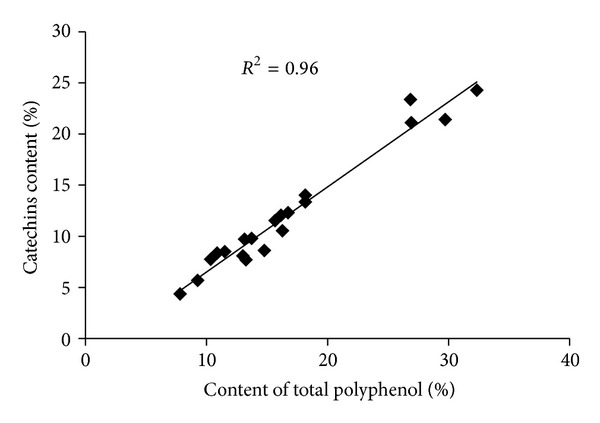
The correlation between total polyphenol and catechin contents.

In addition, the correlation between catechins and total polyphenols in the 20 samples from the four types of tea was tested. The results, shown in Figure 3, reveal a strong positive correlation between catechins and total polyphenols (*R* ^2^ = 0.96).

Table 2 lists the contents of individual catechins in the four types of tea. It is apparent that the content of esterified catechins (EGCG or ECG) is higher than that of nonesterified catechins (catechin, EC, or EGC). In comparison of the four types of tea, EGCG content was the highest in green tea (5.25–14.39% on dry tea) and in oolong tea (4.57–13.08% on dry tea). With the increase in the degree of fermentation, the EGCG content gradually decreased and the range of EGCG content in dark tea is only 0.36–0.93% on dry tea. Among the four types of tea, the ECG content was the highest in black tea (3.38–5.63% on dry tea) and in dark tea (1.92–4.73% on dry tea).

### 3.2. Oxygen Radical Absorbance Capacity (ORAC) in Four Types of Tea

**Figure 4:**
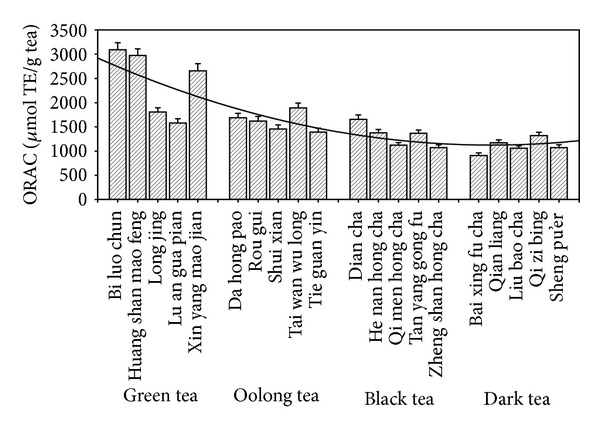
Oxygen radical absorbance capacity (ORAC) values of different samples in four types of tea.

A straight calibration curve was obtained for trolox antioxidant activity in the concentration range of 1.25–80 *μ*M: *y* = 0.36*x* − 0.56, where *x* is concentration of trolox and *y* is Net AUC (*R* ^2^ = 0.9983).  Figure 4 shows the ORAC values of the selected tea samples; the ORAC value ranged from 909.28 to 3092.51 *μ*mol TE/g. Of the selected teas, the antioxidant activity as assessed by the ORAC method decreased in the following order: green tea > oolong tea > black tea > dark tea.* Huang shan mao feng* (green tea) showed the strongest antioxidant activity.

**Figure 5:**
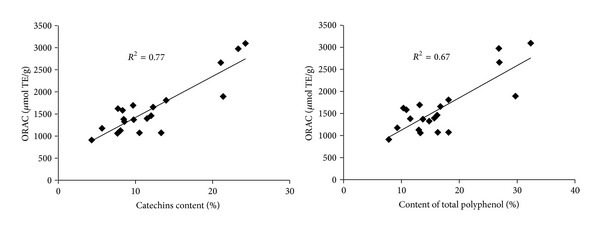
Correlation analysis of ORAC values against total polyphenols and catechins in tea.

Figure 5 shows the relationships between the ORAC value and total polyphenols and catechins, respectively. A positive correlation was observed in each case, with *R* ^2^ = 0.67 for total polyphenols and *R* ^2^ = 0.77 for catechins. In addition, the correlations between individual catechin content and ORAC values in 20 samples were evaluated. The correlations between ORAC value and content of catechin, EGC, EC, EGCG, and ECG, respectively, are shown in Table 3, which shows a positive correlation for each case. In green tea, the EC content showed a relatively higher positive correlation (*R* ^2^ = 0.73) with ORAC values than catechin, EGC, EGCG, and ECG, and the values were significantly different (*P* < 0.01). For black tea, the EGCG content showed a relatively higher positive correlation (*R* ^2^ = 0.82) with ORAC values than catechin, EC, EGC, and ECG, and the values were significantly different (*P* < 0.01). In contrast, the correlation coefficients between ORAC value and content of individual catechin in oolong tea and in dark tea infusions were not significantly different (*P* < 0.01).

### 3.3. DPPH Radical-Scavenging Activity in Four Types of Tea

**Figure 6:**
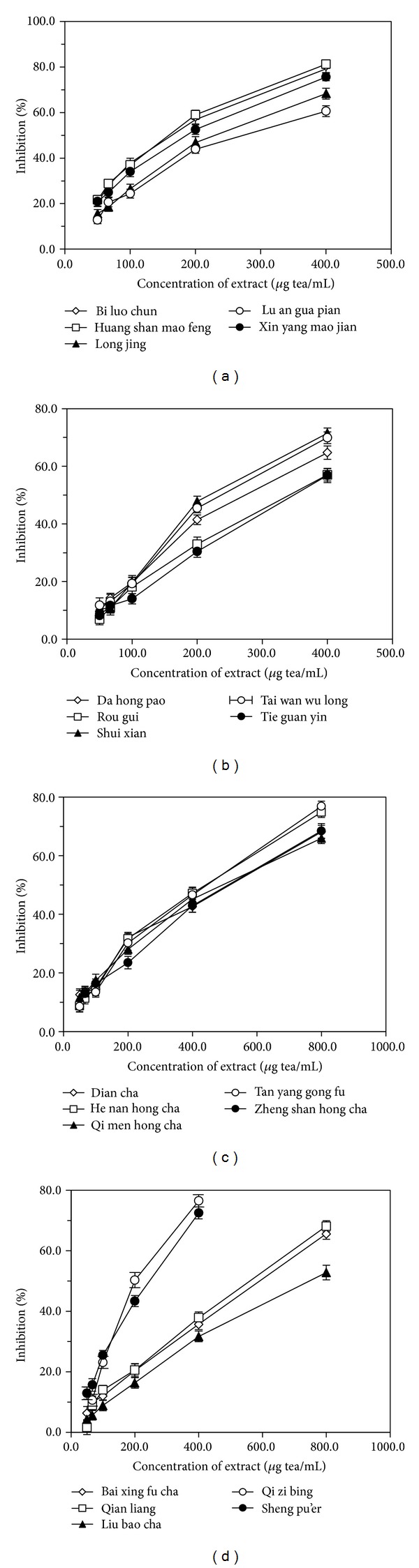
Percentage inhibition of DPPH radical by tea extract for green teas (a), oolong teas (b), black teas (c), and dark teas (d).

The free radical-scavenging activity of the 20 samples of the four types of tea was assessed by DPPH assay; the results are shown in Figure 6. All sample extracts showed good inhibitory activity against the DPPH radical in a dose-dependent manner. The EC50 and AE values of the tea sample extracts are summarized in Table 4, where lower EC50 values or higher AE values suggest higher antioxidant activity. For the 20 selected tea samples, EC50 was in the range 0.142–0.764 mg tea/mL and the AE values ranged from 0.117 to 0.848. Generally, DPPH radical-scavenging efficiency decreased in the following order: green tea > oolong tea > black tea > dark tea. The scavenging efficiency of* Huang shan mao feng* (green tea) against the DPPH radical was the strongest; its EC50 and AE values were 0.142 mg tea/mL and 0.848, respectively.

**Figure 7:**
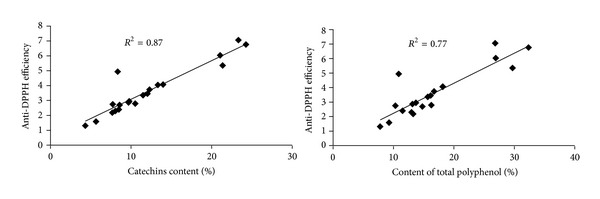
Correlation analysis of anti-DPPH efficiency (AE) values versus total polyphenols and catechins in tea.

Figure 7 shows the respective correlations between the AE values and the content of total catechins and total polyphenols. In each case, good correlation was observed (*R* ^2^ = 0.87 for total catechins; *R* ^2^ = 0.77 for total polyphenols).

The correlations between AE values and the content of individual catechins in the 20 tea infusions are summarized in Table 5; significant positive correlation was observed in each case. Among the individual catechins in green tea, the EGCG content showed a relatively higher positive correlation (*R* ^2^ = 0.86) with AE values than those of catechin, EC, EGC, and ECG, and the values were significantly different (*P* < 0.01).

### 3.4. Online HPLC DPPH Radical-Scavenging Activity of Tea Samples

In the tests of antioxidant capacity in the 20 samples of tea, as measured by the DPPH method, it was not clear which component played the important role in antioxidant activity. The recent development of the online HPLC DPPH method was used to our advantage in this study. This method, which is now used widely in the testing of food and agricultural products and in the pharmaceutical industry [35–37], allows the radical-scavenging activity of a single substance to be measured. This allows the calculation of a component's contribution to the overall radical-scavenging activity of a mixture of antioxidants [37]. Thus, the radical-scavenging activities of the major catechins (EGC, EC, EGCG, ECG, and catechin), gallic acid, and caffeine were determined by the online HPLC DPPH screening method.

**Figure 8:**
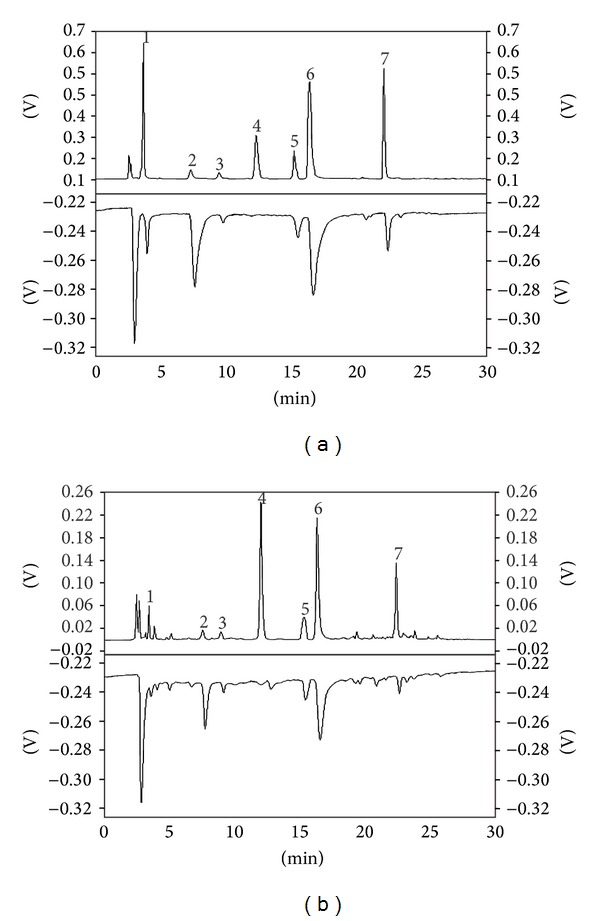
Chromatograms at 278 nm (upper traces) and online DPPH quenching profiles (lower traces) for mixed catechin standards (a) and a tea sample ((b) Bi luo chun). 1: gallic acid; 2: EGC; 3: catechin; 4: caffeine; 5: EC; 6: EGCG; 7: ECG.

**Figure 9:**
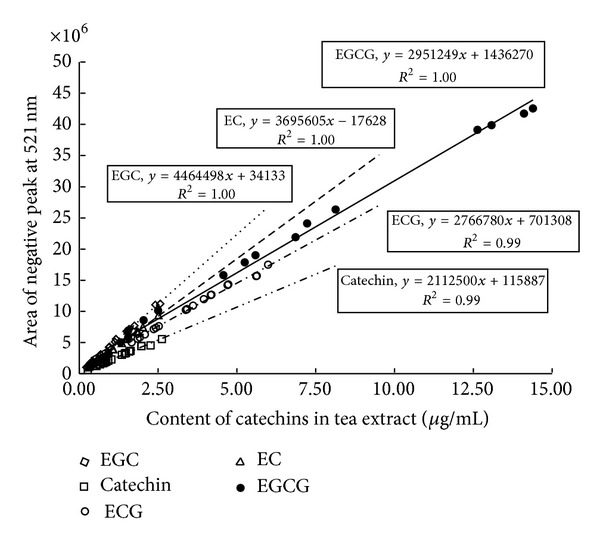
DPPH-scavenging activities of different catechins.

**Figure 10:**
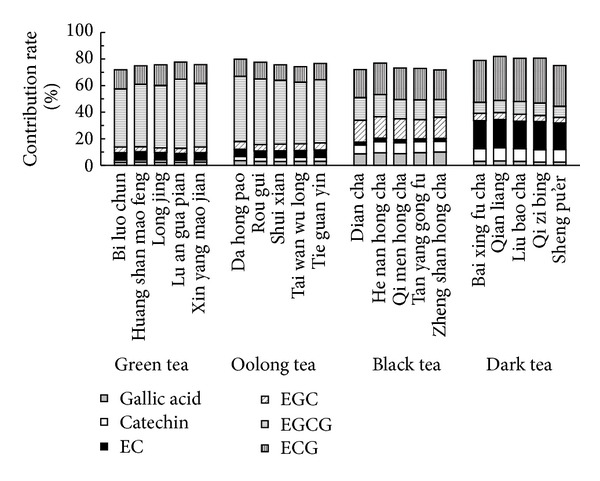
Contributions of gallic acid and catechins in tea to DPPH-scavenging activity.

The chromatograms of the mixed catechin standards and that of a tea sample are shown in Figure 8, illustrating the excellent baseline separation of EGC, EC, EGCG, ECG, catechin, gallic acid, and caffeine. The areas of the positive peaks at 278 nm were used for quantitative analysis of EC, EGC, ECG, EGCG, gallic acid, catechin, and caffeine, while the areas of the negative peaks at 521 nm were used to evaluate the DPPH-scavenging activity. The results showed that while caffeine had almost no DPPH-scavenging activity, strong positive correlations were observed for the contents of the respective HPLC-separated catechins and for gallic acid (see Figure 9). For the same reaction time (1.0 min), the antioxidant activity as assessed by the online DPPH radical-scavenging method decreased in the following order: EGC > gallic acid > EGCG > EC > ECG > catechin. The contributions of gallic acid and the respective catechins in tea to the DPPH-scavenging activity were calculated based on the area ratios of corresponding negative peaks to the total area of all negative peaks at 521 nm; the results are shown in Figure 10. EGCG can be seen as the main contributor to DPPH-scavenging activity in green and oolong teas, while ECG was a significant contributor in black and dark teas. In general, the DPPH-scavenging activity can be mainly attributed to the esterified catechins (EGCG or ECG). In addition to gallic acid and catechins, other compound/s in tea appear to contribute to the DPPH-scavenging activity. Based on the results shown in Figure 10, these minor contributions are in the range of 18.2–28.3%.

## 4. Conclusions

We conducted a systematic study on the total polyphenol and catechins content, as well as antioxidant activity, in four types of tea (green, dark, oolong, and black) prepared from* Camellia sinensis* in China. The highest content of polyphenols and total catechins was detected in green tea, followed by oolong, black, and dark tea. There was a positive correlation between the antioxidant capacity and total polyphenol content or catechins content (*R* ^2^ = 0.67–0.87, *P* < 0.05). In general, it appears that the longer-fermented teas have lower total polyphenol content, lower catechins content, and lower antioxidant capacity. According to our results, green tea in China is of very high quality in terms of antioxidant capacity when compared with the other teas.

Results of online HPLC DPPH radical-scavenging activity tests of tea samples showed that gallic acid, EGCG, ECG, EC, EGC, and catechin were responsible for the antioxidant capacity of the extracts from tea samples. Furthermore, the antioxidant capacity of tea extracts was mainly attributed to the esterified catechins (EGCG or ECG). The results also suggested that other compound/s in tea may also contribute to the antioxidant capacity to a minor degree.

While consumers will always rely on personal preferences relating to color, taste, and aroma when selecting tea, we believe that the antioxidant capacity should also be considered. The results of this study should prove useful to any consumers who wish to select tea based on antioxidant capacity.

## Figures and Tables

**Table 1:** Source and production date of 20 tea samples.

Category	Product name	Source	Production date
Green tea	Bi luo chun	Jiangsu	2010
	Huang shan mao feng	Anhui	2010
	Long jing	Zhejiang	2011
	Lu an gua pian	Anhui	2010
	Xin yang mao jian	Henan	2010

Oolong tea	Da hong pao	Fujian	2010
	Rou gui	Fujian	2010
	Shui xian	Fujian	2010
	Taiwan oolong	Taiwan	2010
	Tie guan yin	Fujian	2010

Black tea	Dian cha	Yunnan	2010
	Henan black tea	Henan	2009
	Qimen black tea	Anhui	2010
	Tanyang gongfu	Fujian	2010
	Zhengshan black tea	Fujian	2010

Dark tea	Bai xing fu cha	Hunan	2009
	Qian liang	Hunan	2008
	Liu bao cha	Guangxi	2007
	Qi zi bing	Yunnan	2007
	Sheng pu'er	Yunnan	2009

**Table 2:** The content of total polyphenols, total catechins, and individual catechins in 20 samples from four types of tea (*n* = 3).

Samples of tea	EGC (%)	Catechin (%)	EC (%)	EGCG (%)	ECG (%)	Nonesterified catechins (%)	Esterified catechins (%)	Total catechins (%)	Total polyphenols (%)
Green tea									
Bi luo chun	1.13 ± 0.06	0.92 ± 0.04	1.84 ± 0.03	14.39 ± 0.41	5.99 ± 0.02	3.89 ± 0.13	20.38 ± 0.43	24.27 ± 0.56	32.36 ± 0.93
Huang shan mao feng	0.94 ± 0.03	0.85 ± 0.04	1.83 ± 0.05	14.11 ± 0.47	5.61 ± 0.04	3.62 ± 0.12	19.72 ± 0.51	23.34 ± 0.63	26.86 ± 0.49
Long jing	0.62 ± 0.02	0.55 ± 0.05	1.08 ± 0.01	8.13 ± 0.39	3.61 ± 0.11	2.25 ± 0.08	11.74 ± 0.50	13.99 ± 0.58	18.17 ± 0.71
Lu an gua pian	0.37 ± 0.01	0.27 ± 0.01	0.61 ± 0.03	5.25 ± 0.26	1.85 ± 0.03	1.25 ± 0.05	7.10 ± 0.29	8.35 ± 0.34	10.88 ± 0.06
Xin yang mao jian	0.96 ± 0.02	0.74 ± 0.03	1.49 ± 0.01	12.63 ± 0.23	5.26 ± 0.14	3.19 ± 0.06	17.89 ± 0.37	21.08 ± 0.43	26.94 ± 0.09
Mean value	0.80 ± 0.03	0.67 ± 0.03	1.37 ± 0.03	10.90 ± 0.35	4.46 ± 0.07	2.84 ± 0.09	15.37 ± 0.42	18.21 ± 0.51	23.04 ± 0.46
Oolong tea									
Da hong pao	0.63 ± 0.02	0.66 ± 0.03	0.74 ± 0.03	5.59 ± 0.22	2.08 ± 0.02	2.03 ± 0.08	7.67 ± 0.24	9.70 ± 0.32	13.16 ± 0.09
Rou gui	0.44 ± 0.02	0.55 ± 0.01	0.52 ± 0.02	4.57 ± 0.09	1.66 ± 0.06	1.51 ± 0.05	6.23 ± 0.15	7.74 ± 0.20	10.35 ± 0.08
Shui xian	0.68 ± 0.01	0.81 ± 0.02	0.90 ± 0.02	7.23 ± 0.31	2.43 ± 0.01	2.39 ± 0.05	9.66 ± 0.32	12.05 ± 0.37	16.18 ± 0.27
Tai wan wu long	1.16 ± 0.02	1.44 ± 0.05	1.53 ± 0.06	13.08 ± 0.27	4.17 ± 0.06	4.13 ± 0.13	17.25 ± 0.33	21.38 ± 0.46	29.73 ± 0.04
Tie guan yin	0.68 ± 0.01	0.77 ± 0.02	0.84 ± 0.02	6.86 ± 0.07	2.36 ± 0.01	2.29 ± 0.05	9.22 ± 0.08	11.51 ± 0.13	15.69 ± 0.73
Mean value	0.72 ± 0.02	0.85 ± 0.03	0.91 ± 0.03	7.47 ± 0.19	2.54 ± 0.03	2.47 ± 0.07	10.01 ± 0.22	12.48 ± 0.30	17.02 ± 0.24
Black tea									
Dian cha	2.41 ± 0.09	2.50 ± 0.04	0.43 ± 0.01	2.26 ± 0.03	4.70 ± 0.15	5.34 ± 0.14	6.96 ± 0.18	12.30 ± 0.32	16.75 ± 0.74
He nan hong cha	1.58 ± 0.03	1.63 ± 0.06	0.31 ± 0.01	1.55 ± 0.02	3.42 ± 0.01	3.52 ± 0.10	4.97 ± 0.03	8.49 ± 0.13	11.53 ± 0.04
Qi men hong cha	1.50 ± 0.03	1.59 ± 0.05	0.28 ± 0.01	1.31 ± 0.05	3.38 ± 0.11	3.37 ± 0.09	4.69 ± 0.16	8.06 ± 0.25	13.01 ± 0.17
Tan yang gong fu	1.75 ± 0.05	1.97 ± 0.04	0.32 ± 0.01	1.55 ± 0.02	4.19 ± 0.13	4.04 ± 0.10	5.74 ± 0.15	9.78 ± 0.25	13.73 ± 0.04
Zheng shan hong cha	2.57 ± 0.09	2.66 ± 0.12	0.45 ± 0.01	2.04 ± 0.07	5.63 ± 0.01	5.68 ± 0.22	7.67 ± 0.08	13.35 ± 0.30	18.17 ± 0.12
Mean value	1.96 ± 0.06	2.07 ± 0.06	0.36 ± 0.01	1.74 ± 0.04	4.26 ± 0.08	4.39 ± 0.13	6.01 ± 0.12	10.40 ± 0.25	14.64 ± 0.22
Dark tea									
Bai xing fu cha	0.22 ± 0.01	0.77 ± 0.01	1.07 ± 0.03	0.36 ± 0.01	1.92 ± 0.03	2.06 ± 0.05	2.28 ± 0.04	4.34 ± 0.09	7.82 ± 0.29
Qian liang	0.28 ± 0.03	1.02 ± 0.04	1.35 ± 0.07	0.50 ± 0.02	2.52 ± 0.05	2.65 ± 0.14	3.02 ± 0.07	5.67 ± 0.21	9.29 ± 0.38
Liu bao cha	0.34 ± 0.01	1.35 ± 0.02	1.87 ± 0.04	0.72 ± 0.02	3.41 ± 0.17	3.56 ± 0.07	4.13 ± 0.19	7.69 ± 0.26	13.28 ± 0.60
Qi zi bing	0.35 ± 0.01	1.48 ± 0.07	2.02 ± 0.07	0.79 ± 0.04	3.95 ± 0.13	3.85 ± 0.15	4.74 ± 0.17	8.59 ± 0.32	14.80 ± 0.03
Sheng pu'er	0.39 ± 0.02	1.97 ± 0.02	2.51 ± 0.09	0.93 ± 0.02	4.73 ± 0.13	4.87 ± 0.13	5.66 ± 0.15	10.53 ± 0.28	16.29 ± 0.17
Mean value	0.32 ± 0.02	1.32 ± 0.03	1.76 ± 0.06	0.66 ± 0.02	3.31 ± 0.10	3.40 ± 0.11	3.97 ± 0.12	7.36 ± 0.23	12.30 ± 0.29

**Table 3:** Correlation coefficients for correlation of ORAC value and catechins content in different tea infusions.

Catechins content	Green tea	Oolong tea	Black tea	Dark tea
EGC (%)	0.53^a^	0.89^a^	0.73^a^	0.68^a^
Catechin (%)	0.66^b^	0.90^a^	0.76^b^	0.66^a^
EC (%)	0.73^c^	0.87^a^	0.76^b^	0.65^a^
EGCG (%)	0.69^d^	0.89^a^	0.82^c^	0.66^a^
ECG (%)	0.65^b^	0.90^a^	0.70^d^	0.69^a^

Values marked by the same lowercase superscript letter within the same column are not significantly different (*P* < 0.01).

**Table 4:** EC50 values (concentration causing 50% inhibition) and anti-DPPH radical efficiency (AE) by tea extract.

Sample of teas	EC50 (mg tea/mL)	AE
Green tea		
Bi luo chun	0.148	0.830
Huang shan mao feng	0.142	0.848
Long jing	0.246	0.609
Lu an gua pian	0.203	0.693
Xin yang mao jian	0.166	0.780
Mean value	0.181	0.761
Oolong tea		
Da hong pao	0.349	0.456
Rou gui	0.365	0.438
Shui xian	0.290	0.538
Tai wan wu long	0.187	0.728
Tie guan yin	0.299	0.525
Mean value	0.298	0.550
Black tea		
Dian cha	0.268	0.572
He nan hong cha	0.418	0.378
Qi men hong cha	0.437	0.360
Tan yang gong fu	0.339	0.470
Zheng shan hong cha	0.247	0.606
Mean value	0.342	0.489
Dark tea		
Bai xing fu cha	0.764	0.117
Qian liang	0.634	0.199
Liu bao cha	0.459	0.339
Qi zi bing	0.372	0.430
Sheng pu'er	0.359	0.444
Mean value	0.518	0.324

**Table 5:** Correlation coefficients between AE and catechins content in different tea infusions.

Catechins content	Green tea	Oolong tea	Black tea	Dark tea
EGC (%)	0.79^a^	0.98^a^	0.99^a^	0.96^ab^
Catechin (%)	0.79^a^	1.00^bc^	1.00^a^	0.94^a^
EC (%)	0.84^b^	0.98^ab^	0.98^ab^	0.96^bc^
EGCG (%)	0.86^c^	1.00^c^	0.91^c^	0.98^c^
ECG (%)	0.79^a^	1.00^c^	0.98^ab^	0.98^c^

Values marked by the same lowercase superscript letter within same column are not significantly different (*P* < 0.01).
